# Mortality and loss to follow-up among HIV-infected persons on long-term antiretroviral therapy in Latin America and the Caribbean

**DOI:** 10.7448/IAS.18.1.20016

**Published:** 2015-07-10

**Authors:** Gabriela Carriquiry, Valeria Fink, John Robert Koethe, Mark Joseph Giganti, Karu Jayathilake, Meridith Blevins, Pedro Cahn, Beatriz Grinsztejn, Marcelo Wolff, Jean William Pape, Denis Padgett, Juan Sierra Madero, Eduardo Gotuzzo, Catherine Carey McGowan, Bryan Earl Shepherd

**Affiliations:** 1 Instituto de Medicina Tropical Alexander von Humboldt, Lima, Peru; 2 Fundación Huésped, Buenos Aires, Argentina; 3 Department of Medicine, Vanderbilt University Nashville, TN, USA; 4 Department of Biostatistics, Vanderbilt University Nashville, TN, USA; 5 Instituto de Pesquisa Clinica Evandro Chagas-Fundação Oswaldo Cruz, Rio de Janeiro, Brazil; 6 Fundación Arriarán, Santiago, Chile; 7 Le Groupe Haïtien d'Etude du Sarcome de Kaposi et des Infections Opportunistes, Port-au-Prince, Haiti; 8 Weill Cornell Medical College, New York, NY, USA; 9 Instituto Hondureño de Seguridad Social and Hospital Escuela, Tegucigalpa, Honduras; 10 Instituto Nacional de Ciencias Médicas y Nutrición, Mexico City, Mexico

**Keywords:** HIV, AIDS, ART, Latin America, the Caribbean, long-term mortality

## Abstract

**Introduction:**

Long-term survival of HIV patients after initiating highly active antiretroviral therapy (ART) has not been sufficiently described in Latin America and the Caribbean, as compared to other regions. The aim of this study was to describe the incidence of mortality, loss to follow-up (LTFU) and associated risk factors for patients enrolled in the Caribbean, Central and South America Network (CCASAnet).

**Methods:**

We assessed time from ART initiation (baseline) to death or LTFU between 2000 and 2014 among ART-naïve adults (≥18 years) from sites in seven countries included in CCASAnet: Argentina, Brazil, Chile, Haiti, Honduras, Mexico and Peru. Kaplan-Meier techniques were used to estimate the probability of mortality over time. Risk factors for death were assessed using Cox regression models stratified by site and adjusted for sex, baseline age, nadir pre-ART CD4 count, calendar year of ART initiation, clinical AIDS at baseline and type of ART regimen.

**Results:**

A total of 16,996 ART initiators were followed for a median of 3.5 years (interquartile range (IQR): 1.6–6.2). The median age at ART initiation was 36 years (IQR: 30–44), subjects were predominantly male (63%), median CD4 count was 156 cells/µL (IQR: 60–251) and 26% of subjects had clinical AIDS prior to starting ART. Initial ART regimens were predominantly non-nucleoside reverse transcriptase inhibitor based (86%). The cumulative incidence of LTFU five years after ART initiation was 18.2% (95% confidence interval (CI) 17.5–18.8%). A total of 1582 (9.3%) subjects died; the estimated probability of death one, three and five years after ART initiation was 5.4, 8.3 and 10.3%, respectively. The estimated five-year mortality probability varied substantially across sites, from 3.5 to 14.0%. Risk factors for death were clinical AIDS at baseline (adjusted hazard ratio (HR)=1.65 (95% CI 1.47–1.87); *p*<0.001), lower baseline CD4 (HR=1.95 (95% CI 1.63–2.32) for 50 vs. 350 cells/µL; *p<*0.001) and older age (HR=1.47 (95% CI 1.29–1.69) for 50 vs. 30 years at ART initiation; *p*<0.001).

**Conclusions:**

In this large, long-term study of mortality among HIV-positive adults initiating ART in Latin America and the Caribbean, overall estimates of mortality were heterogeneous, generally falling between those reported in high-income countries and sub-Saharan Africa.

## Introduction

The HIV epidemic in Latin America and the Caribbean is unique but under-studied. In 2013, the World Health Organization (WHO) estimated that 1.5 million (1.2–1.9 million) people were living with HIV/AIDS in Latin America, and an additional 250,000 (220,000–280,000) in Caribbean countries [1]. In the last decade, rates of new infections in the Caribbean declined more than any other region (49%). However, the Caribbean continues to have the second highest prevalence of HIV infection after sub-Saharan Africa, with an estimated 1% of adults living with HIV, while the estimated prevalence in Latin America is lower at 0.4% [1]. Access to HIV care and highly active antiretroviral therapy (ART) in Latin America and the Caribbean has expanded rapidly over the past decade; of the estimated 910,000 persons in need of ART by 2010 WHO guidelines, 72% in the Caribbean and 75% in Latin America were on ART [1].

Latin America and the Caribbean represent a geographically large area with diverse economic and social contexts, but relatively few studies describing mid- and long-term health outcomes among HIV-infected individuals on ART have included this region [2–6]. A previous analysis incorporating data through 2006 found wide variability in one-year mortality at seven sites in Latin America and the Caribbean, but survival and program retention over longer follow-up periods have not been adequately described [2]. The past decade has seen the rapid global expansion of access to ART to meet the need for treatment, and epidemiologic data on long-term HIV health outcomes are now available to gauge the success of these efforts both within and between regions. In this analysis, we utilized data from the Caribbean, Central and South America Network (CCASAnet) cohort to describe risk factors for mortality and loss to follow-up (LTFU) for up to 14 years after ART initiation among HIV-infected adults.

## Methods

### Cohort description

This observational cohort study included antiretroviral-naïve adults (≥18 years) initiating their first ART regimen on or after 1 January 2000 at nine sites (seven countries) participating in CCASAnet [7]: Hospital Fernández and Centro Médico Huésped in Buenos Aires, Argentina (HF/CMH-Argentina); Instituto de Nacional de Infectologia Evandro Chagas, Fundação Oswaldo Cruz in Rio de Janeiro, Brazil (FIOCRUZ-Brazil); Fundación Arriarán in Santiago, Chile (FA-Chile); Le Groupe Haïtien d'Etude du Sarcome de Kaposi et des Infections Opportunistes in Port-au-Prince, Haiti (GHESKIO-Haiti); Instituto Hondureño de Seguridad Social and Hospital Escuela in Tegucigalpa, Honduras (IHSS/HE-Honduras); El Instituto Nacional de Ciencias Médicas y Nutrición Salvador Zubirán in Mexico City, Mexico (INNSZ-Mexico); and Instituto de Medicina Tropical Alexander von Humboldt in Lima, Peru (IMTAvH-Peru). The two sites in Buenos Aires, Argentina, were grouped together in all analyses as were the two sites in Tegucigalpa, Honduras. The database closing date was 1 January 2013 for HF/CMH-Argentina, GHESKIO-Haiti, and IHSS/HE-Honduras, and 1 January 2014 for the other sites.

### Data management

Clinical, laboratory and demographic data were collected at each centre, de-identified and sent to the CCASAnet Data Coordinating Center at Vanderbilt University (VDCC), Nashville, TN, USA, for data cleaning, processing and merging. The VDCC checked data for internal consistency and performed on-site data audits to verify the accuracy of data received with that contained in the medical record [8]. Institutional ethics review boards from each participating site and Vanderbilt University reviewed and approved the project. Informed consent process was done at IMTAvH-Peru and waived on all the other sites.

### Outcomes and study definitions

The primary endpoint was all-cause mortality. Different methods were used to ascertain death across sites. At GHESKIO-Haiti and IHSS/HE-Honduras, death was recorded when field workers were notified by family members after a call due to patients missing a visit. At all other sites, relatives of patients informed staff of the death (unless it occurred, and was already recorded, at the hospital), and in addition, study staff checked at least annually for subjects LTFU in government death registry databases for the FIOCRUZ-Brazil, FA-Chile, INNSZ-Mexico and IMTAvH-Peru sites. The exact date of death was unknown for 6% (98/1582) of deaths. Patients whose date of death was known up to the month (*n*=13) were arbitrarily assigned a date of death as the 15th day of the month; those whose date of death was known up to the year (*n*=5) were assigned a date of death of June 15. Those whose date of death was known to occur before/after a given date (*n*=2/*n*=78) were assigned a date of death two months before/after the given date; two months was chosen as an approximate midpoint between study visits. Because the percentage of patients with a missing date of death was quite small, these choices had little impact on results.

LTFU was defined as no clinical visit within the year preceding database closing date and unknown vital status. Subjects who were known to have transferred to another clinic were not counted as LTFU. Clinical stage prior to initiation of first ART was categorized as AIDS or not AIDS; clinical AIDS was defined as CDC stage C (FIOCRUZ-Brazil, FA-Chile, IHSS/HE-Honduras and INNSZ-Mexico), WHO stage IV (GHESKIO-Haiti, IMTAvH-Peru) or a specification of AIDS at first visit (HF/CMH-Argentina). GHESKIO-Haiti classified WHO stages II and III into a single category, which prohibited sensitivity analyses defining AIDS as WHO stage III or IV. Nadir CD4 at ART initiation was defined as the lowest measured CD4 count prior to or no more than seven days after initiating ART. Plasma HIV-1 RNA level (viral load, VL) at ART initiation was defined as the measurement closest to initiating ART but no more than 180 days before; any viral load measurement after initiating was not included.

### Statistical analysis

The probability of mortality after ART initiation was estimated using Kaplan-Meier methods, censoring subjects who were LTFU at the time of their last visit. The cumulative incidence of LTFU after ART initiation was estimated by site treating death as a competing risk. We performed an additional analysis estimating the probability of mortality accounting for LTFU/censoring by fitting a Cox proportional hazards model (described below) using patient characteristics at ART initiation, estimating the predicted survival probability over time per patient from that model, and then averaging across patients at each site. By incorporating baseline characteristics at ART initiation, this analysis attempts to account for potential bias due to differential LTFU.

Risk factors at ART initiation for subsequent mortality were assessed using Cox proportional hazards models. All models (unadjusted and adjusted) were stratified by CCASAnet site. The primary adjusted models included sex, calendar year, age, CD4 count, clinical AIDS at ART initiation and ART regimen class. Secondary analyses also included pre-ART viral load and probable route of infection (heterosexual, homosexual, injection drug use (IDU) or other/missing); GHESKIO-Haiti did not measure viral load or collect probable route of infection over much of the study period and was, therefore, not included in analyses incorporating viral load. The adjusted analyses used multiple imputations via a chained equations approach (package “mi” in R statistical software) with 10 imputation replications to account for missing data. Age, square-root-transformed CD4 count, calendar year and log-transformed viral load were included in the models using restricted cubic splines with four knots to relax linearity assumptions. Predicted five-year survival probabilities as a function of each variable were computed from the fitted model holding all other variables constant at representative levels. To account for potentially time-dependent associations between baseline predictors and mortality, we fit a separate Cox model limiting follow-up to the first year, then we fit a second Cox model limiting follow-up only to the second year among those who lived through the first year, then we fit a third Cox model limiting follow-up only to the third year among those who lived through the second year, and so forth up to seven years. This analysis attempts to obtain estimates in a setting where the proportional hazards assumption may be violated. All analyses were performed using R Statistical Software (www.R-project.org); analysis scripts are available at biostat.mc.vanderbilt.edu/ArchivedAnalyses.

## Results

### Patient characteristics

**Figure 1:**
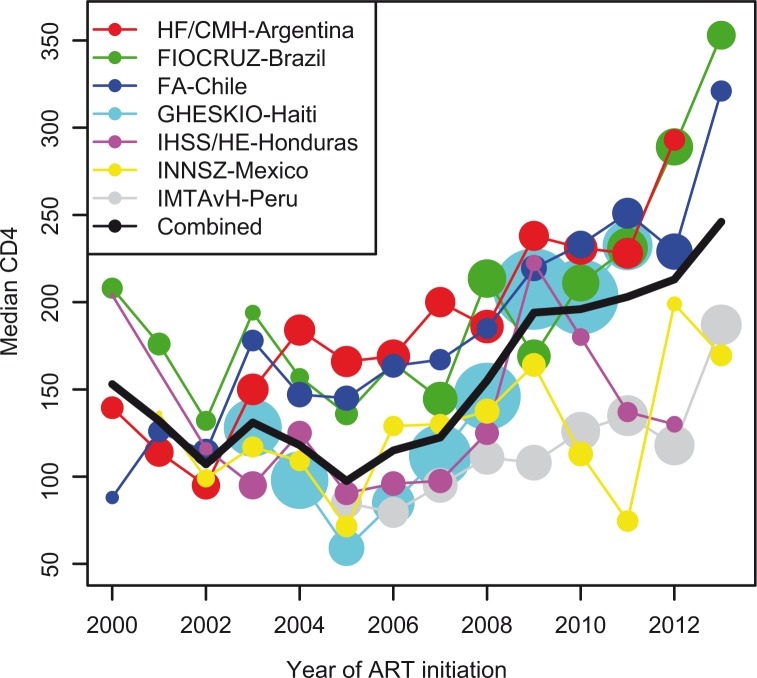
CD4 at ART initiation. The radius of each circle is proportional to the number of patients starting ART for a given site and year.

A total of 16,996 antiretroviral-naïve subjects initiating ART were included in this study: 2408 (14%) from HF/CMH-Argentina, 2273 (13%) from FIOCRUZ-Brazil, 1697 (10%) from FA-Chile, 6468 (38%) from GHESKIO-Haiti, 960 (6%) from IHSS/HE-Honduras, 1010 (6%) from INNSZ-Mexico and 2180 (13%) from IMTAvH-Peru. Patient characteristics at ART initiation by site are given in Table 1. ART initiators were predominantly male (63%) ranging from 44% in GHESKIO-Haiti to 89% in FA-Chile. The median age at ART initiation was 36 years (interquartile range (IQR): 30–44 years). Twenty-six percent of subjects had clinical AIDS prior to starting ART. The median pre-ART CD4 count was 156 cells/µl (IQR: 60–251 cells/µl), but this increased with calendar year; trends in CD4 at ART initiation for each site are shown in Figure 1. The median year of ART initiation was 2008, and the vast majority of subjects (86%) started a regimen containing a non-nucleoside reverse transcriptase inhibitor (NNRTI).

**Table 1:** Summary of patient characteristics by site at ART initiation

	HF/CMH-Argentina	FIOCRUZ-Brazil	FA-Chile	GHESKIO-Haiti	IHSS/HE-Honduras	INNSZ-Mexico	IMTAvH-Peru	Combined

	(*n*=2408)	(*n*=2273)	(*n*=1697)	(*n*=6468)	(*n*=960)	(*n*=1010)	(*n*=2180)	(*n*=16,996)
Sex								
Female	722 (30.0%)	676 (29.7%)	183 (10.8%)	3617 (55.9%)	429 (44.7%)	112 (11.1%)	627 (28.8%)	6366 (37.5%)
Male	1686 (70.0%)	1597 (70.3%)	1514 (89.2%)	2851 (44.1%)	531 (55.3%)	898 (88.9%)	1553 (71.2%)	10,630 (62.5%)
Probable route of infection								
Heterosexual	959 (39.8%)	1075 (47.3%)	415 (24.5%)	0 (0.0%)	565 (58.9%)	289 (28.6%)	1387 (63.6%)	4690 (27.6%)
Homo/bisexual	678 (28.2%)	816 (35.9%)	1266 (74.6%)	0 (0.0%)	65 (6.8%)	673 (66.6%)	771 (35.4%)	4269 (25.1%)
IDU	173 (7.2%)	15 (0.7%)	4 (0.2%)	0 (0.0%)	1 (0.1%)	10 (1.0%)	0 (0.0%)	203 (1.2%)
Other	23 (1.0%)	20 (0.9%)	5 (0.3%)	0 (0.0%)	3 (0.3%)	13 (1.3%)	19 (0.9%)	83 (0.5%)
Unknown	575 (23.9%)	347 (15.3%)	7 (0.4%)	6468 (100%)	326 (34.0%)	25 (2.5%)	3 (0.1%)	7751 (45.6%)
Age, years	36 (30, 44)	36 (29, 44)	35 (30, 42)	38 (31, 45)	36 (29, 42)	33 (28, 41)	33 (27, 41)	36 (30, 44)
Clinical stage								
AIDS	534 (22.2%)	161 (7.1%)	476 (28.0%)	1530 (23.7%)	389 (40.5%)	504 (49.9%)	801 (36.7%)	4395 (25.9%)
Not AIDS	1152 (47.8%)	1870 (82.3%)	839 (49.4%)	4930 (76.2%)	544 (56.7%)	401 (39.7%)	1026 (47.1%)	10,762 (63.3%)
Unknown	722 (30.0%)	242 (10.6%)	382 (22.5%)	8 (0.1%)	27 (2.8%)	105 (10.4%)	353 (16.2%)	1839 (10.8%)
Nadir CD4 Pre-ART, cells/µL	184 (74, 271)	209 (79, 304)	182 (71, 268)	150 (62, 234)	115 (58, 195)	124 (38, 240)	115 (45, 231)	156 (60, 251)
Missing	300 (12.5%)	206 (9.1%)	267 (15.7%)	281 (4.3%)	143 (14.9%)	151 (15.0%)	75 (3.4%)	1423 (8.4%)
VL, pre-ART (copies/mL×1000)	69 (14, 210)	71 (16, 223)	93 (22, 280)	–	91 (27, 100)	75 (48, 117)	148 (53, 350)	89 (25, 256)
Missing	801 (33.3%)	506 (22.3%)	504 (29.7%)	6468 (100.0%)	817 (85.1%)	194 (19.2%)	558 (25.6%)	9848 (57.9%)
Undetectable	71 (2.9%)	26 (1.1%)	14 (0.8%)	0 (0.0%)	7 (0.7%)	17 (1.7%)	0 (0.0%)	135 (0.8%)
Year of initial regimen	2006 (2003, 2009)	2009 (2006, 2011)	2007 (2003, 2011)	2008 (2005, 2009)	2006 (2004, 2008)	2008 (2005, 2010)	2010 (2007, 2012)	2008 (2005, 2010)
Initial regimen class								
NNRTI	1652 (68.6%)	1614 (71.0%)	1400 (82.5%)	6205 (95.9%)	916 (95.4%)	794 (78.6%)	2059 (94.4%)	14640 (86.1%)
Boosted PI	568 (23.6%)	475 (20.9%)	183 (10.8%)	190 (2.9%)	18 (1.9%)	180 (17.8%)	112 (5.1%)	1726 (10.2%)
Unboosted PI	84 (3.5%)	131 (5.8%)	98 (5.8%)	9 (0.1%)	24 (2.5%)	16 (1.6%)	4 (0.2%)	366 (2.2%)
Other	104 (4.3%)	53 (2.3%)	16 (0.9%)	64 (1.0%)	2 (0.2%)	20 (2.0%)	5 (0.2%)	264 (1.6%)

Continuous variables reported with median (interquartile range). ART: highly active antiretroviral therapy; HF/CMH-Argentina: Hospital Fernandez and Centro Médico Huesped, Buenos Aires, Argentina; FIOCRUZ-Brazil: Instituto de Nacional de Infectologia Evandro Chagas, Fundação Oswaldo Cruz, Rio de Janeiro, Brazil; FA-Chile: Fundación Arriarán, Santiago, Chile; GHESKIO-Haiti: Le Groupe Haïtien d'Etude du Sarcome de Kaposi et des Infections Opportunistes, Port-au-Prince, Haiti; IHSS/HE-Honduras: Instituto Hondureño de Seguridad Social and Hospital Escuela, Tegucigalpa, Honduras; INNSZ-Mexico: El Instituto Nacional de Ciencias Médicas y Nutrición Salvador Zubirán, Mexico City, Mexico; IMTAvH-Peru: Instituto de Medicina Tropical Alexander von Humboldt, Lima, Peru; IDU: injection drug use; VL: plasma HIV-1 RNA level; NNRTI: non-nucleoside reverse transcriptase inhibitor; PI: protease inhibitor.

### Follow-up

The median years of follow-up after ART initiation was 3.5 years (IQR: 1.6–6.2 years) with the longest median follow-up of 4.9 years at FA-Chile and the lowest of 3.0 years at IMTAvH-Peru.

**Figure 2:**
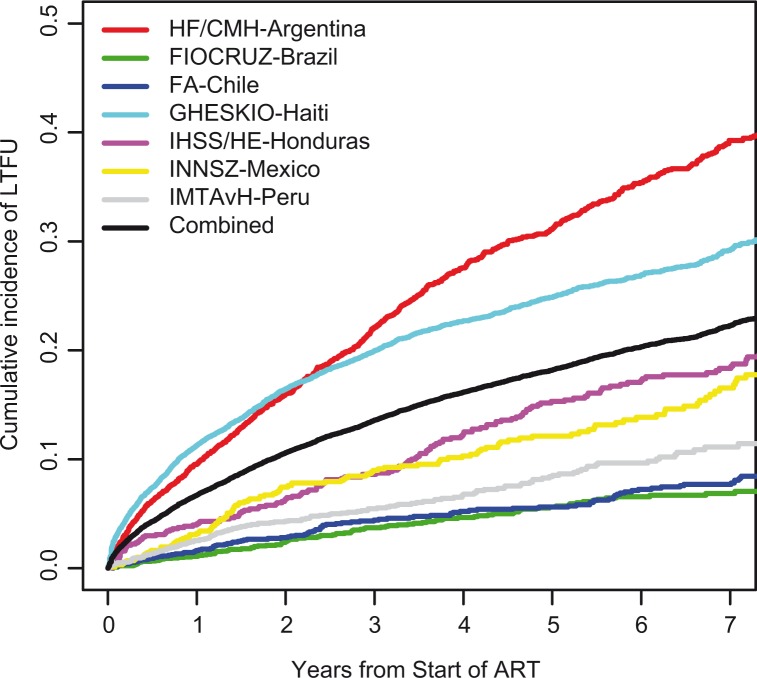
Cumulative incidence of loss to follow-up by site.

The cumulative incidence of LTFU five years after ART initiation was 18.2% (95% confidence interval (CI) 17.5–18.8%) for the combined cohort and is shown in Figure 2. After five years, the cumulative incidence of LTFU was 31.2, 5.6, 5.6, 24.9, 15.3, 12.1 and 8.4% for HF/CMH-Argentina, FIOCRUZ-Brazil, FA-Chile, GHESKIO-Haiti, IHSS/HE-Honduras, INNSZ-Mexico and IMTAvH-Peru, respectively.

Subjects who were LTFU tended to be younger at ART initiation than those remaining in care at all sites except FIOCRUZ-Brazil (*p*=0.82) and FA-Chile (*p*=0.19; *p*<0.05 for all other sites). In HF/CMH-Argentina, patients who were LTFU tended to be in more advanced stages of disease at ART initiation than those who remained in care, but much less advanced stages compared to those who subsequently died (median CD4=197, 166, and 50 cells/µL for alive, LTFU, and dead, respectively; proportion with AIDS=18, 27, and 55%, respectively). Similar trends were seen at GHESKIO-Haiti (median CD4=164, 142 and 83 cells/µL for alive, LTFU and dead, respectively; proportion with AIDS=20, 24 and 44%, respectively). In contrast, at IHSS/HE-Honduras, subjects who were LTFU tended to have lower rates of clinical AIDS prior to starting ART. (Full comparisons between baseline characteristics of subjects LTFU, remaining in care, and dying are provided in the Supplementary file).

### Mortality

**Figure 3:**
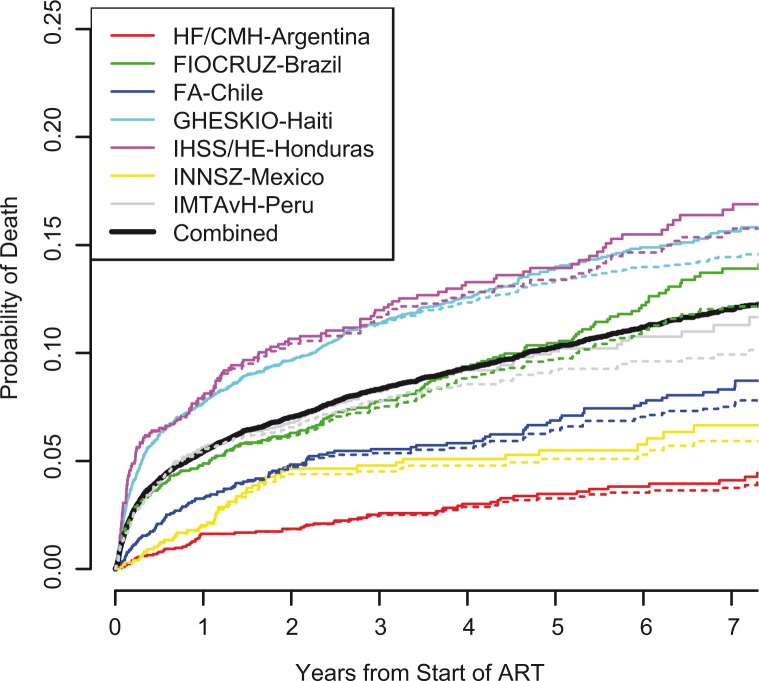
Probability of mortality from start of ART by site. The solid lines represent the Kaplan-Meier estimates, which assume the rate of death among those LTFU/censored is the same as it is among those remaining in follow-up. The dashed lines are the estimated average predicted probabilities based on patient characteristics at ART initiation, which account for differences between baseline characteristics of those LTFU/censored and those remaining in follow-up.

The overall probability of death one, three and five years after initiating ART was 5.4% (95% CI 5.1–5.8), 8.3% (95% CI 7.9–8.8) and 10.3% (95% CI 9.8–10.8), respectively, although estimates were very heterogeneous across sites. For example, year five mortality probabilities were: 3.5, 10.5, 6.9, 14.0, 13.9, 5.5 and 10.1% for HF/CMH-Argentina, FIOCRUZ-Brazil, FA-Chile, GHESKIO-Haiti, IHSS/HE-Honduras, INNSZ-Mexico and IMTAvH-Peru, respectively. Figure 3 shows the estimated probability of mortality over time after ART initiation. The overall incidence of mortality was 22.2 per 1000 person-years (py), although it was high during the first year (57.8 deaths per 1000 py, ranging from 16.4 deaths per 1000 py in HF/CMH-Argentina to 87.4 per 1000 py in IHSS/HE-Honduras) and dropped to 12.6 deaths per 1000 py after the first year (ranging from 5.4 in HF/CMH-Argentina to approximately 16–17 per 1000 py in FIOCRUZ-Brazil, GHESKIO-Haiti and IHSS/HE-Honduras). Figure 3 also shows the average predicted probability of mortality per site, accounting for patient characteristics in those who were LTFU and censored; estimates were similar but slightly lower (The Supplementary file contains a data animation demonstrating survival and LTFU over by time by patient characteristics at ART initiation).

**Figure 4:**
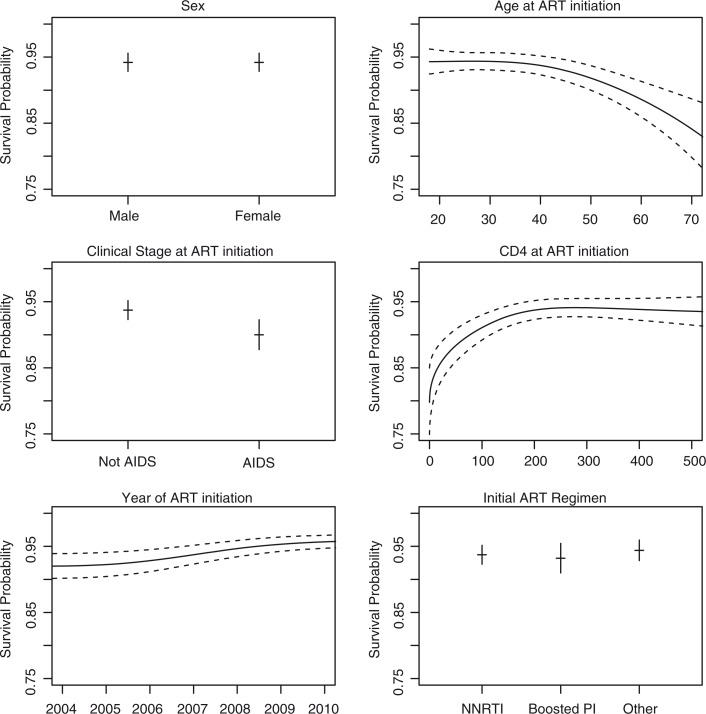
The impact of each variable on the predicted five-year survival probability. The predictive model is adjusted for the following variables (set at the corresponding representative levels): sex (male), age (40 years), clinical stage at baseline (not AIDS), baseline CD4 (200 cells/μL), initial ART regimen (NNRTI-based) and site (Peru).

The relative hazards of mortality after ART initiation, both crude and adjusted, for several patient characteristics are given in Table 2; the association between patient characteristics and the predicted survival probability five years after ART initiation is shown in Figure 4. Clinical AIDS at ART initiation (adjusted hazard ratio (HR)=1.65; 95% CI 1.47–1.87), lower CD4 (adjusted HR=1.95 for 50 vs. 350 cells/µL; 95% CI 1.63–2.32) and older age (adjusted HR=1.47 for 50 vs. 30 years; 95% CI 1.29–1.69) were all associated with higher rates of mortality. After controlling for other variables, subjects initiating ART in more recent years tended to have a lower hazard of mortality (adjusted HR=0.74 for 2008 vs. 2006; 95% CI 0.67–0.82). In unadjusted analyses, male sex was associated with an increased rate of mortality, but this was not seen after controlling for other variables.

**Table 2:** Hazard ratios for mortality after ART initiation

	Unadjusted		Adjusted	
	
	HR (95% CI)	*p*	HR (95% CI)	*p*
Male	1.21 (1.09, 1.35)	<0.001	1.08 (0.97, 1.20)	0.16
Age (years)		<0.001		<0.001
20	0.84 (0.66, 1.06)		1.01 (0.79, 1.28)	
30	1		1	
40 (ref)	1.11 (1.00, 1.24)		1.12 (1.01, 1.24)	
50	1.40 (1.22, 1.60)		1.47 (1.29, 1.69)	
60	1.92 (1.65, 2.23)		2.08 (1.79, 2.42)	
AIDS before ART	2.38 (2.14, 2.66)	<0.001	1.65 (1.47, 1.87)	<0.001
Nadir pre-ART CD4 (cells/µL)		<0.001		<0.001
50	2.65 (2.24, 3.13)		1.95 (1.63, 2.32)	
100	1.87 (1.59, 2.21)		1.49 (1.25, 1.76)	
200	1.09 (0.94, 1.25)		1.05 (0.91, 1.21)	
350 (ref)	1		1	
Year of starting ART		<0.001		<0.001
2000	0.96 (0.73, 1.26)		1.02 (0.77, 1.34)	
2002	1.08 (0.92, 1.26)		1.08 (0.92 1.26)	
2004	1.08 (0.92, 1.26)		1.11 (1.05, 1.18)	
2006 (ref)	1		1	
2008	1.16 (1.10, 1.23)		0.74 (0.67, 0.82)	
2010	0.63 (0.57, 0.69)		0.59 (0.51, 0.68)	
Initial regimen		0.005		0.08
NNRTI (ref)	1		1	
Boosted PI	1.21 (1.02, 1.43)	0.025	1.09 (0.92, 1.30)	0.34
Other	0.91 (0.77, 1.07)	0.25	0.89 (0.75, 1.06)	0.19

Analyses are stratified by study site. ART: highly active antiretroviral therapy; HR: hazard ratio; CI: confidence interval; NNRTI: non-nucleoside reverse transcriptase inhibitor; PI: protease inhibitor.

The association between nadir CD4 and mortality weakened with longer follow-up. Comparing those starting ART with CD4=50 vs. 350 cells/µL, during the first year of ART the adjusted HR=2.86 (95% CI 2.23–3.67); among those who survived the first year, during the second year of ART the adjusted HR=2.56, 95% CI 1.50–4.38. However, among those who survived the first two years, during the third year of ART the adjusted HR=1.26 (95% CI 0.77–2.08), and similar analyses in subsequent years yielded non-significant adjusted HR estimates ranging between 0.70 and 1.08.

We performed secondary analyses excluding GHESKIO-Haiti, as it was an outlier in terms of the patient population, geography and sample size; in addition, viral load was not measured and probable route of HIV infection was not routinely collected at GHESKIO-Haiti. Similar to the primary analyses, AIDS at ART initiation (adjusted HR=1.49; 95% CI 1.24–1.78), lower CD4 count (adjusted HR=1.86 for 50 vs. 350 cells/µL; 95% CI 1.45–2.38) and older age (adjusted HR=1.44 for 50 vs. 30 years; 95% CI 1.19–1.74) were strongly associated with an increased hazard of death. Heterosexually infected men had significantly higher mortality than heterosexually infected women (adjusted HR=1.26; 95% CI 1.05–1.50). Year of ART initiation was also associated with a higher hazard of mortality (adjusted HR=0.89 for 2008 vs. 2006; 95% CI 0.79–1.01). Men whose probable route of infection was homosexual sex had lower rates of mortality than those whose probable route was heterosexual sex (adjusted HR=0.71; 95% CI 0.60–0.86); those infected through IDU tended to have a higher hazard of mortality than those infected through heterosexual sex (adjusted HR=1.63; 95% CI 0.98–2.70). Details of these secondary analyses are given in Table 3. Analyses were repeated, also excluding IHSS/HE-Honduras in which 85% of viral load measurements were missing; results were similar.

**Table 3:** Hazard ratios for mortality after ART initiation after excluding GHESKIO-Haiti

	Unadjusted		Adjusted	
	
	HR (95% CI)	*p*	HR (95% CI)	*p*
Male	1.24 (1.05, 1.46)	0.008	1.26 (1.05, 1.50)	0.011
Age (years)		<0.001		<0.001
20	0.69 (0.48, 0.98)		0.81 (0.57, 1.16)	
30	1		1	
40 (ref)	1.15 (0.99, 1.34)		1.13 (0.97, 1.32)	
50	1.47 (1.22, 1.77)		1.44 (1.19, 1.74)	
60	2.04 (1.66, 2.52)		1.98 (1.60, 2.45)	
AIDS before ART	2.09 (1.77, 2.47)	<0.001	1.49 (1.24, 1.78)	<0.001
Nadir pre-ART CD4 (cells/µL)		<0.001		<0.001
50	2.54 (2.02, 3.18)		1.86 (1.45, 2.38)	
100	1.78 (1.43, 2.22)		1.41 (1.12, 1.77)	
200	1.04 (0.90, 1.20)		1.00 (0.86, 1.17)	
350 (ref)	1		1	
Year of starting ART		<0.001		0.033
2000	1.06 (0.78, 1.44)		1.00 (0.73, 1.38)	
2002	1.08 (0.92, 1.28)		1.04 (0.87 1.23)	
2004	1.08 (0.92, 1.28)		1.05 (0.98, 1.12)	
2006 (ref)	1		1	
2008	1.08 (1.01, 1.15)		0.89 (0.79, 1.01)	
2010	0.84 (0.75, 0.96)		0.79 (0.65, 0.96)	
Initial regimen		0.003		0.039
NNRTI (ref)	1		1	
Boosted PI	1.14 (0.94, 1.39)	0.19	1.09 (0.89, 1.33)	0.43
Other	0.81 (0.67, 0.98)	0.026	0.84 (0.70, 1.02)	0.08
Probable route of infection		<0.001		<0.001
Heterosexual (ref)	1		1	
Homosexual	0.69 (0.58, 0.81)	<0.001	0.71 (0.60, 0.86)	<0.001
IDU	2.04 (1.24, 3.34)	0.005	1.63 (0.98, 2.70)	0.058
Other/Missing	1.21 (0.98, 1.48)	0.076	1.08 (0.88, 1.34)	0.46
Pre-ART log_10_-VL		<0.001		0.69
3	1.50 (1.08, 2.09)		0.87 (0.65, 1.18)	
4	1.50 (1.21, 1.86)		0.97 (0.82, 1.14)	
5 (ref)	1		1	
6	0.77 (0.62, 0.96)		1.04 (0.88, 1.24)	

Analyses are stratified by study site; ART: highly active antiretroviral therapy; GHESKIO-Haiti: Le Groupe Haïtien d'Etude du Sarcome de Kaposi et des Infections Opportunistes, Port-au-Prince, Haiti; HR: hazard ratio; CI: confidence interval; NNRTI: non-nucleoside reverse transcriptase inhibitor; PI: protease inhibitor; IDU: injection drug use; VL: plasma HIV-1 RNA level.

## Discussion

Countries in Latin America and the Caribbean have responded to the HIV epidemic with a robust expansion of ART to serve an estimated 1.6 million HIV-infected individuals, but there are comparatively few data on long-term HIV treatment outcomes compared to other geographic regions [1].

Approximately 10% of patients starting ART were reported deceased at five years, but there was considerable heterogeneity across sites, with the lowest mortality at the site in Argentina and the highest at sites in Haiti and Honduras. Mortality early after ART initiation was particularly high, at nearly 60 deaths per 1000 person years during the first year. Rates of LTFU also varied among sites, with just over two-thirds of patients at the sites in Argentina still engaged in care after five years as compared to 94% at sites in Brazil and Chile.

To our knowledge, this analysis represents the first, large multi-cohort study of long-term mortality and treatment retention among HIV-infected, ART-treated individuals in Latin America. Prior analyses of mortality at one year of ART in the CCASAnet network found an overall mortality of 8.3% (95% CI 7.6–9.1%) over the period 1996–2007, which also varied across sites (2.6–13%) [2]. This was higher than our finding of 5.4% at one year, which may be attributed to the higher CD4 counts at ART initiation, indicative of less severe disease, in later years of the cohort, or improvements in clinical care.

The high degree of variability in mortality and LTFU estimates across CCASAnet sites likely reflects differences in the underlying demographics of the HIV epidemic in the region, local social and economic characteristics, and varying levels of mortality ascertainment. A relatively greater proportion of the Haitian population was affected by the HIV epidemic, and at an earlier time, as compared with other countries in CCASAnet, and the medical response in Haiti developed in the context of greater poverty, political instability, and, more recently, a severe earthquake [9–11]. On the other hand, per capita gross domestic product in Chile in 2014 was approximately half the average in the European Union and among the highest in Latin America, and a prior analysis of long-term outcomes in the Chilean AIDS cohort found survival probabilities similar to the United States and Europe [4,12]. Differences in mortality ascertainment also likely played a role in the heterogeneous mortality rates. It is notable that the four sites that review national death registries are the four sites with the lowest LTFU rates. In addition, the Brazilian site follows an extensive algorithm for patient tracking and has achieved very low rates of LTFU [13]. The Argentine sites are particularly noteworthy, with the highest incidence of LTFU and the lowest incidence of mortality. Although the high rate of LTFU in Argentina may be related to its fragmented health system with patients seeking attention at other sites not included in the cohort [2], the high rate of LTFU may also be related to under-ascertainment of mortality.

It may be useful to compare our estimates of mortality in Latin America and the Caribbean to those from other regions. A recent multiregional study found crude rates of mortality after four years of ART of 4.7, 16.6 and 15.3% in cohorts in Europe, South Africa and North America, respectively [14]; our estimates are much higher than those in Europe but slightly lower than those reported in South Africa and North America, perhaps due to some under-ascertainment of mortality in our setting. Our estimates (mortality incidence of 22 per 1000 person-years after median of 3.5 years of follow-up with median CD4 of 156 cells/µL at baseline) are very similar to those from a recent large study of the national treatment program in Botswana (mortality incidence of mortality of 27 per 1000 person years after median of 2.9 years with median CD4 of 151 cells/µL at baseline) [15]. The Trans-Caribbean HIV/AIDS Research Initiative (TCHARI) group previously reported a 13% three-year mortality incidence in seven Caribbean countries, which was quite similar to our estimated three-year mortality incidence of 12.4% at GHESKIO-Haiti (which is not surprising given that GHESKIO-Haiti accounted for more than half of the patients in the TCHARI study), but higher than our estimate combining data from all cohorts [16]. Our mortality estimates are similar or lower than those in separate studies reported in South Africa [17,18]. Even within similar regions/nations, mortality estimates have been seen to widely vary [19,20]. However, in general terms, the long-term incidence of mortality among those in Latin America and the Caribbean appears to fall somewhere between that of Europe/North America and sub-Saharan Africa, which is consistent with what has been seen during the first year of ART [6].

Risk factors associated with mortality in our study were lower CD4 count, older age and clinical AIDS at ART initiation, which have been reported in other regional settings and a large meta-analysis of 50 studies from low- and middle-income countries in Asia, Africa, and Central and South America [6,21,22]. In our secondary analysis (excluding GHESKIO-Haiti), heterosexually infected males had a higher risk of mortality than heterosexually infected females, which has also been observed in other settings [6,22–24]. The role of low body mass index (BMI) as a risk factor for early ART mortality could not be assessed, as most CCASAnet sites do not record longitudinal BMI. However, previous studies from sub-Saharan Africa, the Caribbean and other regions have identified BMI and body weight as important factors in survival, and we speculate that a similar effect was present in our cohort [6,16,25,26].

At nearly all sites, younger patients were more likely to be LTFU, a result that is consistent with the findings in many other settings [24,25]. At HF/CMH-Argentina and GHESKIO-Haiti, our sites with the highest incidence of LTFU, patients who were lost tended to have stages of disease at ART initiation that were slightly more advanced than those who remained alive and in care, but much less advanced than those who died. These findings differ somewhat from what was observed in an earlier study using data from the same cohort, where in sites from Argentina patients lost during the first year of ART tended to be among the healthiest at ART initiation [2]. However, the trends reported here are consistent with what has been observed in other developing country settings [25]. At our other sites, no such trends were detected. Our mortality estimates that accounted for differential censoring using patient characteristics at ART initiation were actually slightly lower than standard estimates at each site (Figure 3), likely because younger patients and those starting in more recent years, both good predictors of survival, were more likely to be censored.

A strength of this study was the large number of subjects contributing data from multiple countries. A major limitation was the high rates of LTFU at some sites, which may have led to under-reporting of mortality events. Studies tracing patients LTFU in sub-Saharan Africa have discovered high rates of mortality [27–29]. However, it is unlikely that results based on LTFU tracing studies in sub-Saharan Africa apply to patient populations in Latin America. Ascertainment of death varied by site, as some clinics searched government death registry databases, whereas no such databases existed for other sites.

Furthermore, uncertainty in the date of death for a small percentage (6%) of patients who died introduced a degree of imprecision regarding survival time, although this would be expected to have less of an effect on analyses of long-term outcomes (e.g., five years) as compared to short-term outcomes. Other limitations are due to the retrospective nature of our data; for example, clinical AIDS may be slightly different across sites because of heterogeneity in the way clinical stage of disease was captured. Lastly, we were unable to evaluate the role of ART adherence on outcomes, or evaluate differences in the causes of death at study sites or over time. Similar to an earlier study performed at our site in Brazil [30], we suspect the majority of early deaths in our cohort were AIDS-related; however, we are unable to verify this with our data.

In conclusion, this analysis found that long-term mortality and programme retention of HIV-infected adults starting ART in Latin American and the Caribbean over the past decade generally fell between those reported in high-income countries and sub-Saharan Africa, though a high degree of heterogeneity between sites was observed. Further studies are needed to understand the programmatic and large social, economic and health sector factors underlying these differences. As ART treatment programs mature, future studies should evaluate the impact of earlier ART initiation on long-term outcomes in Latin America and the Caribbean, including cost-effectiveness, and the incidence and prevalence of non-communicable diseases among patients on therapy.

## Competing interests

The authors declare that they have no competing interests.

## Authors' contributions

GC, VF, JRK, MJG, KJ, MB, PC, BG, MW, JWP, DP, JS, EG, CCM and BES conceived and designed the study. GC, VF, PC, MW, DP, JWP, JS and EG performed data abstraction. KJ performed data management. GC, VF, JRK, MJG and BES analyzed the data. MB performed data animation. GC, VF, JRK, MJG and BES wrote the draft manuscript. GC, VF, JRK, MJG, KJ, MB, PC, BG, MW, JWP, DP, JS, EG, CCM and BES did the critical appraisal of the manuscript. All authors approved the final version of the manuscript.

## Supplementary Material

Mortality and loss to follow-up among HIV-infected persons on long-term antiretroviral therapy in Latin America and the Caribbean
