# Supplementary material for: Mortality and loss to follow-up among HIV-infected persons on long-term antiretroviral therapy in Latin America and the Caribbean
Source: J Int AIDS Soc. 2015 Jul 10;18(1):20016. doi: 10.7448/IAS.18.1.20016 (PMC4499577; doi:10.7448/IAS.18.1.20016)
Supplement: Mortality and loss to follow-up among HIV-infected persons on long-term antiretroviral therapy in Latin America and the Caribbean [file JIAS-18-20016-s001.pdf]

Supplemental Material

Table 1: Characteristics of Patients LTFU, Alive, and Dead in HF/CMH-Argentina

	Alive (1427)	LTFU (901)	Dead (80)	p-value (Alive vs. LTFU)	p-value (Dead vs. LTFU)
Age (years)	37 (31, 45)	35 (29, 41)	38 (31, 45)	<0.001	0.004
Nadir pre-ART CD4	197 (86, 280)	166 (69, 264)	50 (17, 140)	0.002	<0.001
Year of Starting ART	2007 (2004, 2010)	2005 (2002, 2008)	2003 (2001, 2006)	<0.001	0.003
Male	984 (69%)	638 (70.8%)	64 (80%)	0.37	0.11
Clinical AIDS before ART	250 (17.5%)	240 (26.6%)	44 (55%)	0.001	<0.001
Initial Regimen				<0.001	0.17
NNRTI	1041 (73%)	569 (63.2%)	42 (52.5%)		
Boosted PI	292 (20.5%)	248 (27.5%)	28 (35%)		
Other	94 (6.6%)	84 (9.3%)	10 (12.5%)		

Table 2: Characteristics of Patients LTFU, Alive, and Dead in FIOCRUZ-Brazil

	Alive (1909)	LTFU (124)	Dead (240)	p-value (Alive vs. LTFU)	p-value (Dead vs. LTFU)
Age (years)	36 (29, 43)	34 (29, 43)	39 (31, 47)	0.82	0.005
Nadir pre-ART CD4	214 (86, 308)	199 (84, 301)	123 (44, 249)	0.8	0.005
Year of Starting ART	2009 (2007, 2011)	2008 (2006, 2010)	2006 (2003, 2009)	<0.001	0.001
Male	1331 (69.7%)	94 (75.8%)	172 (71.7%)	0.18	0.47
Clinical AIDS before ART	124 (6.5%)	10 (8.1%)	27 (11.2%)	0.35	0.5
Initial Regimen				0.61	0.83
NNRTI	1373 (71.9%)	84 (67.7%)	157 (65.4%)		
Boosted PI	389 (20.4%)	29 (23.4%)	57 (23.8%)		
Other	147 (7.7%)	11 (8.9%)	26 (10.8%)		

Table 3: Characteristics of Patients LTFU, Alive, and Dead in FA-Chile

	Alive (1426)	LTFU (139)	Dead (132)	p-value (Alive vs. LTFU)	p-value (Dead vs. LTFU)
Age (years)	35 (30, 42)	33 (28, 41)	38 (32, 48)	0.19	0.001
Nadir pre-ART CD4	192 (80, 272)	167 (79, 256)	70 (28, 183)	0.33	<0.001
Year of Starting ART	2008 (2004, 2011)	2005 (2002, 2009)	2004 (2002, 2006)	<0.001	0.039
Male	1282 (89.9%)	120 (86.3%)	112 (84.8%)	0.24	0.86
Clinical AIDS before ART	379 (26.6%)	36 (25.9%)	61 (46.2%)	0.8	<0.001
Initial Regimen				<0.001	0.13
NNRTI	1179 (82.7%)	116 (83.5%)	105 (79.5%)		
Boosted PI	163 (11.4%)	6 (4.3%)	14 (10.6%)		
Other	84 (5.9%)	17 (12.2%)	13 (9.8%)		

Table 4: Characteristics of Patients LTFU, Alive, and Dead in GHESKIO-Haiti

	Alive (4064)	LTFU (1648)	Dead (756)	p-value (Alive vs. LTFU)	p-value (Dead vs. LTFU)
Age (years)	38 (31, 45)	36 (30, 44)	40 (32, 47)	<0.001	<0.001
Nadir pre-ART CD4	164 (73, 248)	142 (58, 219)	83 (29, 166)	<0.001	<0.001
Year of Starting ART	2009 (2006, 2010)	2008 (2005, 2009)	2006 (2004, 2008)	<0.001	<0.001
Male	1736 (42.7%)	751 (45.6%)	364 (48.1%)	0.052	0.26
Clinical AIDS before ART	799 (19.7%)	398 (24.2%)	333 (44%)	<0.001	<0.001
Initial Regimen				0.38	0.002
NNRTI	3896 (95.9%)	1592 (96.6%)	717 (94.8%)		
Boosted PI	126 (3.1%)	44 (2.7%)	20 (2.6%)		
Other	42 (1%)	12 (0.7%)	19 (2.5%)		

Table 5: Characteristics of Patients LTFU, Alive, and Dead in IHSS/HE-Honduras

	Alive (645)	LTFU (172)	Dead (143)	p-value (Alive vs. LTFU)	p-value (Dead vs. LTFU)
Age (years)	36 (30, 43)	34 (27, 40)	38 (30, 42)	0.017	0.007
Nadir pre-ART CD4	125 (62, 200)	118 (60, 214)	75 (45, 155)	1	0.002
Year of Starting ART	2006 (2004, 2009)	2006 (2004, 2008)	2005 (2003, 2007)	0.014	0.019
Male	360 (55.8%)	82 (47.7%)	89 (62.2%)	0.069	0.014
Clinical AIDS before ART	282 (43.7%)	55 (32%)	52 (36.4%)	0.008	0.37
Initial Regimen				0.021	0.9
NNRTI	620 (96.1%)	161 (93.6%)	135 (94.4%)		
Boosted PI	14 (2.2%)	2 (1.2%)	2 (1.4%)		
Other	11 (1.7%)	9 (5.2%)	6 (4.2%)		

Table 6: Characteristics of Patients LTFU, Alive, and Dead in INNSZ-Mexico

	Alive (815)	LTFU (142)	Dead (53)	p-value (Alive vs. LTFU)	p-value (Dead vs. LTFU)
Age (years)	33 (28, 41)	32 (27, 38)	36 (29, 44)	0.02	0.003
Nadir pre-ART CD4	129 (41, 249)	114 (36, 222)	44 (22, 115)	0.32	0.002
Year of Starting ART	2009 (2006, 2011)	2004 (2003, 2007)	2005 (2004, 2008)	<0.001	0.33
Male	725 (89%)	123 (86.6%)	50 (94.3%)	0.51	0.21
Clinical AIDS before ART	392 (48.1%)	68 (47.9%)	44 (83%)	0.77	<0.001
Initial Regimen				0.09	0.37
NNRTI	653 (80.1%)	106 (74.6%)	35 (66%)		
Boosted PI	138 (16.9%)	27 (19%)	15 (28.3%)		
Other	24 (2.9%)	9 (6.3%)	3 (5.7%)		

Table 7: Characteristics of Patients LTFU, Alive, and Dead in IMTAvH-Peru

	Alive (1853)	LTFU (149)	Dead (178)	p-value (Alive vs. LTFU)	p-value (Dead vs. LTFU)
Age (years)	33 (27, 41)	30 (25, 36)	36 (29, 45)	<0.001	<0.001
Nadir pre-ART CD4	123 (48, 239)	120 (54, 211)	51 (18, 115)	0.99	<0.001
Year of Starting ART	2010 (2008, 2012)	2009 (2007, 2011)	2009 (2007, 2011)	<0.001	0.53
Male	1323 (71.4%)	98 (65.8%)	132 (74.2%)	0.17	0.13
Clinical AIDS before ART	646 (34.9%)	49 (32.9%)	106 (59.6%)	0.89	<0.001
Initial Regimen				<0.001	0.012
NNRTI	1758 (94.9%)	130 (87.2%)	171 (96.1%)		
Boosted PI	87 (4.7%)	18 (12.1%)	7 (3.9%)		
Other	8 (0.4%)	1 (0.7%)	0 (0%)		

Abbreviations used: LTFU: lost to follow-up; ART: highly active antiretroviral therapy; HF/CMHArgentina:Hospital Fernandez and Centro Médico Huesped, Buenos Aires, Argentina; FIOCRUZ-Brazil:Instituto de Nacional de Infectologia Evandro Chagas, Fundacao Oswaldo Cruz, Rio de Janeiro, Brazil;FA-Chile: Fundación Arriarán, Santiago, Chile; GHESKIO-Haiti: Le Groupe Haïtien d'Etude du Sarcomede Kaposi et des Infections Opportunistes, Port-au-Prince, Haiti; IHSS/HE-Honduras: Instituto Hondureño de Seguridad Social and Hospital Escuela, Tegucigalpa, Honduras; INNSZ-Mexico: El Instituto Nacional de Ciencias Medicas y Nutrición Salvador Zubirán, Mexico City, Mexico; IMTAvH-Peru: Instituto de MedicinaTropical Alexander von Humboldt, Lima, Peru; NNRTI: non-nucleoside reverse transcriptase inhibitor; PI:protease inhibitor.
